# Association of Typical Toxic Heavy Metals with Schizophrenia

**DOI:** 10.3390/ijerph16214200

**Published:** 2019-10-30

**Authors:** Jiahui Ma, Lailai Yan, Tongjun Guo, Siyu Yang, Chen Guo, Yaqiong Liu, Qing Xie, Jingyu Wang

**Affiliations:** 1 Department of Laboratorial Science and Technology, School of Public Health, Peking University, Beijing 100191, China; mjh@bjmu.edu.cn (J.M.); yll@bjmu.edu.cn (L.Y.); chenpreteen@sina.com (C.G.); liuyaqiong@bjmu.edu.cn (Y.L.); xieqing94@bjmu.edu.cn (Q.X.); 2 Vaccine Research Center, School of Public Health, Peking University, Beijing 100191, China; 3 Peking University Medical and Health Analysis Center, Peking University, Beijing 100191, China; 4 Department of Occupational and Environmental Health Sciences, School of Public Health, Peking University, Beijing 100191, China; tongjun@bjmu.edu.cn; 5 Tianjin Center for Disease Control and Prevention, No. 6, Huayue Road, Hedong District, Tianjin 300011, China; sy_yang1120@163.com

**Keywords:** toxic heavy metals, chromium, lead, schizophrenia, serum, risk

## Abstract

Toxic heavy metals (THMs) are contaminants commonly found in the environment. Although a large number of studies have demonstrated their damage to the biological functions of the human being, their potential associations with the risk of developing schizophrenia remain controversial. In this study, we investigated the associations between four THMs (chromium (Cr), cadmium (Cd), lead (Pb) and arsenic (As)) in serum and the risk of schizophrenia. In total, 95 patients with schizophrenia (cases) and 95 normal subjects (controls) were recruited from Hebei Province, China. The serum concentrations of the 4 THMs were analyzed by inductively coupled plasma mass spectrometry (ICP-MS). A higher concentration of Pb was found significantly associated with an elevated risk of schizophrenia (OR = 3.146; 95%CI: 1.238–7.994, *p* = 0.016), while significant association for the other three THMs were not observed. Besides, significant correlations were found between the metabolic biomarkers and the concentrations of Pb and As, respectively. In order to further characterize the association between these THMs and schizophrenia with greater statistical power, we conducted meta-analysis by including 538 cases and 1040 controls from the current study and 5 available datasets published from 2002 to 2018. Using a random-effect model, Cr was significantly associated with schizophrenia (SMD = 0.3246; 95%CI: 0.0166–0.6326, *p* < 0.01). Overall, this study suggested that higher levels of Pb and Cr may be one of the factors associated with an elevated risk of schizophrenia.

## 1. Introduction

Schizophrenia is a severe mental illness that affects about one percent of the world’s population [1]. The patients with schizophrenia are characterized with cognitive impairment, confusion, hallucinations and deterioration of social function [2]. It is noted that schizophrenia leads to a heavy burden on the patients, their families and society [2]. Substantial efforts have been devoted to elucidating the pathophysiology of schizophrenia development from genetic and environmental perspectives, as well as gene-environment interactions [3]. Epidemiological studies have suggested that urban environments could increase the risk of schizophrenia [4,5], which may be due to the exposure to the toxic heavy metals (THMs) in the environment.

Chronic exposure to THMs impairs numerous biological functions and results in a series of health problems [6], such as aberrant immunological responses and neuropathological conditions [7,8]. Moreover, it has been reported that THMs could cause oxidative stress and subsequently DNA damage, lipid peroxidation and protein modification, which play a role in the pathogenesis of numerous diseases, including cancer, diabetes, neurological disorders and chronic inflammation [9]. THMs are suggested as an important risk factor for mental diseases since they cause neurobehavioral alterations and disrupt dopamine receptors [10]. For example, arsenic (As) -induced neurotoxicity has been reported to cause compositional changes and hyperphosphorylation on the cytoskeletal protein, leading to the disintegration of the cytoskeletal framework and causing neurodegenerative diseases [11]. An animal study showed lead (Pb) as an environmental toxin, which contributed to the pathogenesis of schizophrenia in Disrupted-in-Schizophrenia 1 (Disc1)-mutant mice [12]. Therefore, investigating the association of environmental THM exposure with the risk of schizophrenia via a reliable epidemiological study is worthwhile. 

The serum concentration of THMs has been suggested to act as an internal indicator of their intake levels [13,14], reflecting exposure to environmental THMs. Changes in THM concentrations have been observed in peripheral tissues to be correlated with the risk of schizophrenia [15]. Although multiple studies have investigated the association between THMs and schizophrenia, the results remain inconsistent. For example, despite supporting evidence both in vitro and in vivo for the association between Pb and schizophrenia [16,17], a cross-sectional study among a Chinese population showed no significant difference in the concentration of Pb between patients with schizophrenia and healthy controls [18]. However, another study applying a very similar study design towards the same sampling region resulted in a significant association [13]. The reason for the divergence may be related to the choice of the population, such as whether the schizophrenic patients have received antipsychotics drug during the time of study [13,16,18,19,20]. A previous study has compared serum cadmium (Cd) concentration between healthy controls and schizophrenia patients with or without treatment. The serum concentration of Cd in patients after treatment was significantly higher than that of the healthy controls but no significant change was observed from the pre-treatment patients [20]. The concentration of antimony and uranium were also found significantly different between pre-treatment patients and post-treatment patients of schizophrenia [15]. 

Although several studies have investigated the association between THMs and schizophrenia, the potential mechanisms which could link THMs and schizophrenia have not been elucidated. Recent studies have suggested metabolic syndrome as a risk factor of schizophrenia, attributed to poor dietary habits, unhealthy lifestyle and physical inactivity of the patients [21] and side-effects of the second generation-antipsychotics [22]. On the other hand, THMs such as As [23] and Pb [24] were found to be associated with metabolic diseases, suggesting a role of abnormal metabolisms in linking THMs and schizophrenia.

Therefore, we performed a case-control study with first-episode and drug-naïve schizophrenia patients and healthy controls to identify the association between serum concentrations of the 4 THMs (i.e., Cr, Cd, Pb and As, selected as commonly surveyed heavy metals in schizophrenia studies) and the risk of schizophrenia [13,16,18,19,20]. The correlations between the 4 THMs and multiple metabolic biomarkers were also tested to explore the potential metabolisms associated between these THMs and schizophrenia. Furthermore, meta-analysis was conducted to comprehensively summarize the association between the 4 individual THMs and the risk of schizophrenia. 

## 2. Methods

### 2.1. Participants

All participants are permanent residents in Tangshan, Hebei Province, China. The patients with schizophrenia were recruited from the Tangshan Psychiatric Hospital from May 2018 to May 2019. All the patients who were admitted to the hospital during this period were screened by internal medicine specialists. Patients were recruited by the following criteria—(1) meet the schizophrenia diagnostic criteria according to the International Classification of Diseases 10th Revision (no. F20). (2) first-episode and drug-naïve and (3) without another psychiatric disorder besides schizophrenia. Age-sex matched healthy controls without any known psychiatric problems were recruited in the same period in the Tangshan Workers’ Hospital. The inclusion criteria applied for all subjects were as follows—(1) 18‒60 years of age, (2) no history of occupational exposure in the heavy industry, (3) no acute infectious and traumatic diseases, (4) no evidence of chronic physical illness and (5) not receiving mineral or vitamin supplements that might have influenced metal levels. These conditions were consulted by internal medicine specialists. Ultimately, this study recruited 95 cases and 95 controls.

Demographic information was surveyed through face-to-face interviews. These include age (“<25”, “25–30”, “30–35”, “>35”), sex (“male”, “female”), BMI (“<18.5”, “18.5–24.9”, “25–29.9”, “>30”), marital status (“single”, “married”), education level (“elementary school or below”, “junior high school”, “high school”, “college or above”), active smoking (“no”, “yes”) and drinking (“no”, “yes”). Results of blood biochemical tests, including glucose metabolism (i.e., fasting blood glucose (FBG)), lipid metabolism (i.e., triglycerides (TG) and total cholesterol (TC)), liver function, i.e., aspartate transaminase (AST), alanine transaminase (ALT), albumin (ALB) and total protein (TP)) and renal function (i.e., blood urea nitrogen (BUN), creatinine (CREA) and uric acid (UA)), were performed and provided by the hospital. The Positive and Negative Syndrome Scale (PANSS) score for patients was determined by at least four professional physicians based on their clinical experience. The study protocol was approved in May 24th of 2018 by the Ethics Review Committee of the Health Science Center, Peking University (IRB00001052-18028). Written consent was obtained from all participants. 

### 2.2. Sample Collection and ICP Analysis

Blood sample (~3 mL) was collected from subjects after an overnight fasting and stored in a metal-free plastic tube. All blood samples were kept at room temperature for 30 min and then centrifuged at 3000 rpm for 15 min. Serum sample was transferred to a centrifuge tube and stored at −80 °C until all the samples were collected. For the measurement of the 4 THMs (Cr, Cd, Pb and As), serum sample (0.1 mL) was mixed with 0.1 mL indium (2 ng/mL) as internal standard element, followed by 1.8 mL 1% nitric acid. The concentrations of the 4 THMs were then measured by inductively coupled plasma mass spectrometry (ICP-MS, ELAN DRCII, PerkinElmer, USA). ClinChek® Serum Controls (trace elements level II, REF 8881, EVISA) and Human Hair Certified Reference Material (GBW09101b, China) were used as standards for quality control. The measurements were based on the most abundant isotope of each element to avoid interference. The detection limit of the 4 THMs by this analytical method, the analytical results of the Certified Reference Material (CRM) and reproducibility of the CRM measurement were showed in Tables S1 and S2. The protocol was qualified by the China Metrology Accreditation (CMA) system (160018001523). The laboratory has participated in national-level evaluation and comparison and achieved “satisfactory” results. All the experimental staff of this study have received professional training for this test.

### 2.3. Statistical Analysis

Chi-Squared (χ2) and Mann-Whitney U tests were used for analyzing the difference of categorical variable and continuous variable respectively. An unconditional logistic regression model was used to analyze the associations between serum THM levels and the risk of schizophrenia. Variables significantly different between case and control (i.e., marital status) was adjusted in the model. Odds ratios (ORs) and 95% confidence intervals (95% CIs) were calculated. A two-tailed *p* value < 0.05 was considered as statistical significance. Spearman correlation was used to calculate the correlation coefficients (rho) between THMs and metabolic biomarkers. All statistical analyses were performed using SPSS ver. 21.0 (SPSS Inc., Chicago, IL, USA).

### 2.4. Meta-Analysis

Meta-analysis was conducted from the studies which have measured the 4 THMs in serum of schizophrenia patients and controls. These studies were found by searching PubMed, Excerpta Medica dataBASE, the Cochrane Databases, China National Knowledge Infrastructure, SinoMed and Wanfang database from inception till June 30, 2019. The principles of meta-analysis from Cochrane Library (i.e., PICOS (participants, interventions, comparisons, outcomes and study design)) [25] were used for literature searching and formulating the inclusion and exclusion criteria. Studies passed the following three criteria were included—(1) being a case-control study, (2) using standard diagnostic criteria for schizophrenia patient diagnosis and (3) having the quality score of the Newcastle-Ottawa Quality Assessment Scale (NOS) above 6 [26].

Data extraction was conducted by two independent curators, including sample size, study area, whether the patients with schizophrenia used anti-schizophrenia drugs, the mean concentration of THMs and the standard deviation of THMs for patients with schizophrenia and healthy controls. Any discrepancy was discussed and resolved by consensus by one extra curator.

In the meta-analysis, random effect model and standardized mean difference (SMD) with 95% CI were used to evaluate the association between the THMs and schizophrenia risk. We used Cochran’s Q and *I*^2^ statistic to estimate the heterogeneity amongst studies. Statistically significant heterogeneity was defined as a *p* < 0.1 or *I*^2^ > 50%; Whether the patients had received antipsychotic drugs during the study period (i.e., drug = 1/ drug = 0) were used for subgroup analysis. Sensitivity analysis was performed to strengthen the reliability of the result. All data analyses were performed using R Version 3.6.0 software (R Foundation for Statistical Computing, Vienna, Austria, 2019).

## 3. Results

### 3.1. Description of Subjects and Serum THM Concentrations

In this study, 95 schizophrenic patients and 95 healthy subjects without schizophrenia were recruited. The distributions of the demographic characteristics of the cases and controls are presented in Table 1. Significant difference was observed only from the marital status, while no significant differences was found in the other characteristics (i.e., age, sex, BMI, education level, active smoking and drinking status).

We focused on 4 THMs (i.e., Cr, Cd, Pb and As) in the serum of the subjects. Among these 4 THMs, serum concentration of As was significantly lower in the patient group than the control group (*p* < 0.05), while concentration of Pb was significant higher in the patient group than the control group (Table 2). No significant difference between patients and controls was found for Cr and Cd. All THMs analyzed in this study showed 100% detection rates.

### 3.2. Associations between THM Concentrations and Schizophrenia

We calculated the ORs associated with the risk of schizophrenia for the 4 THMs using continuous serum metal concentrations. Analysis based on unconditional logistic regression models demonstrated that the concentration of Pb was associated with the risk of schizophrenia with or without adjustment for potential confounders (i.e., marital status) (Table 3, adjusted OR = 3.212; 95% CI: 1.322–7.805; *p* = 0.016). The concentrations of Cr, Cd and As were not associated with the risk of schizophrenia. To investigate the relationship between THM concentrations and schizophrenia severity, we further analyzed the correlation between the concentration of THMs and PANSS scores. The results suggested that the concentration of Pb and PANSS scores (i.e., total and negative scores) were positively correlated (Table 4). 

### 3.3. Correlation between THM Concentrations and Metabolic Biomarkers

Ten metabolic biomarkers were chosen to compare the metabolic conditions between patients with schizophrenia and healthy controls. Significant differences were observed from FBG, TC, ALT, ALB, TP and BUN. Spearman correlation was applied to determine the association between THMs and metabolic conditions (i.e., glucose metabolism, lipid metabolism, liver and renal function) (Table 5). A significant negative correlation (*p* < 0.05) was found between the serum concentration of Pb and ALB (rho = −0.163), BUN (rho = −0.182), CREA (rho = −0.207) and UA (rho = −0.170). A significant positive correlation (*p* < 0.05) was found between the concentration of As and TC (rho = 0.186), ALB (rho = 0.163), TP (rho = 0.208) and BUN (rho = 0.217). There was no significant correlation between the other THMs and metabolic biomarkers. 

### 3.4. Meta-Analysis—Associations between THM Concentrations and Schizophrenia

**Figure 1:**
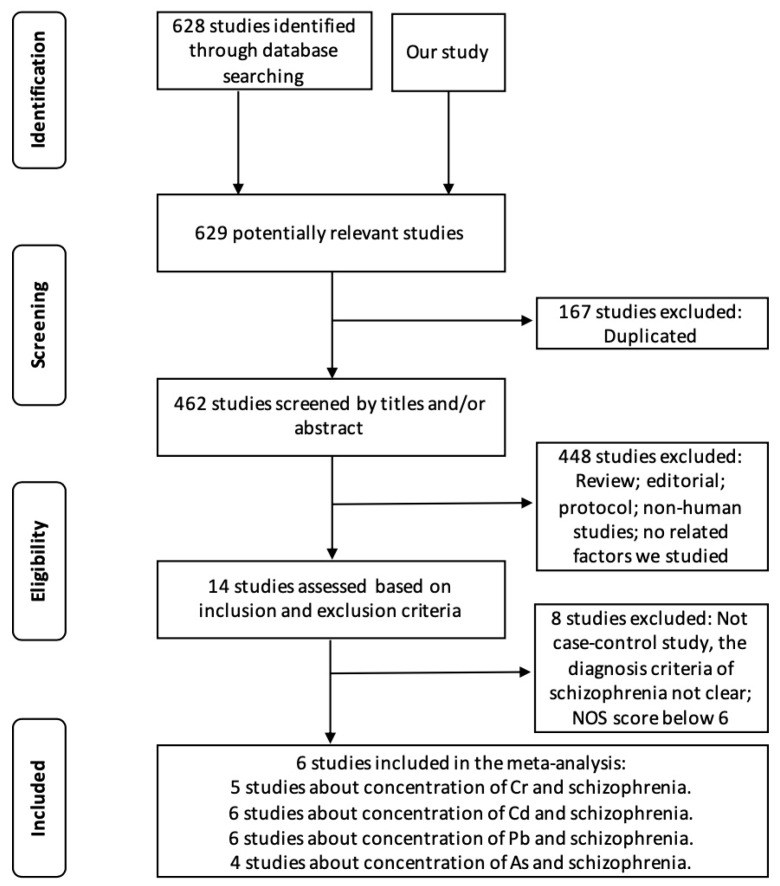
Flow diagram of the studies selection process for the present meta-analysis.

**Figure 2:**
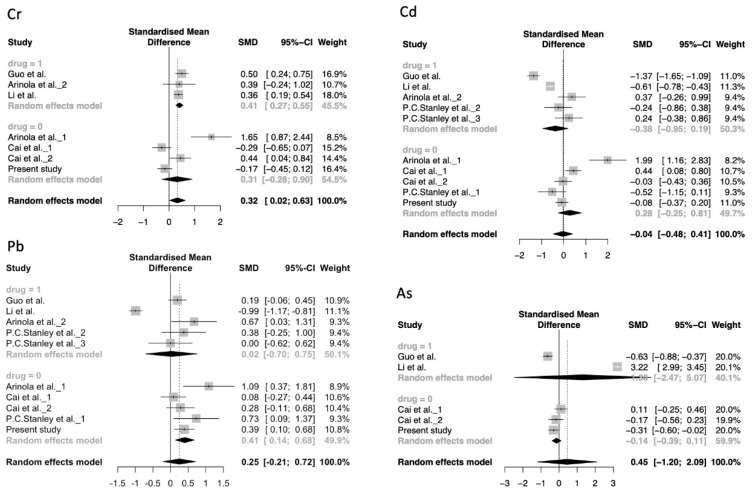
Forest plot of overall meta-analysis and subgroup analysis of the association between the concentration of each THM and schizophrenia. Overall pooled prevalence was calculated by random-effects module. drug = 1, patients with schizophrenia were treated with antipsychotic drugs. drug = 0, patients with schizophrenia were not treated with antipsychotic drugs. Horizontal bars show 95% CIs.

Starting from 629 identified records, 6 studies (538 schizophrenia cases and 1040 controls) were included for analysis. The selection process and result of study inclusion were showed in Figure 1 and Table S3 respectively. Our meta-analysis suggested a significant association of higher level of Cr with schizophrenia risk (Figure 2 and Table S4, SMD = 0.41; 95% CI: 0.10–0.73). As the overall results of the four THMs were heterogeneous (*I*^2^ > 50%, *p* < 0.1) (Table S4), we further performed subgroup analysis based on whether the patients have received antipsychotic drugs during the study period (Figure 2). Significantly, an association was found in the subgroup of Cr that patients have received antipsychotic drugs (SMD = 0.41; 95% CI: 0.27–0.55) as well as in the subgroup of Pb patients that have not received antipsychotic drugs (SMD = 0.41; 95% CI: 0.14–0.68). Moreover, for Pb, the result remains the same if we withdraw our dataset (Figure S1, SMD = 0.45; 95% CI: 0.06–0.85). To determine the effect of an individual dataset to the pooled ORs, sensitivity analysis was conducted and the results were consistent in all of the research models for Cd and As (Figure S2).

## 4. Discussion

Higher concentrations of serum Pb were significantly associated with an elevated risk of schizophrenia in our independent study with drug-naïve schizophrenia patients and the meta-analysis under the subgroup condition that only drug-free patients are considered. Significant association between Cr and schizophrenia was also shown in the meta-analysis. 

Lead is one of the environmental toxic substances characterized by its neurotoxic potential [27]. Direct, as well as chronic, exposure to Pb has been widely reported to cause severe neurological symptoms and cognitive impairment [27]. A follow-up study from the Child Health and Development Study birth cohort has suggested that prenatal Pb exposure may increase the risk of developing schizophrenia later in life [28]. Meanwhile, in a multidecade longitudinal study of Pb-exposed children, higher blood Pb level in childhood was associated with greater psychopathology along the life span and difficult different adult personality traits [29], highlighting the neurotoxic role of Pb in childhood. Results of the present study indicated an association between serum concentration of Pb and schizophrenia. This result is echoed by our meta-analysis in the subgroup of drug-free schizophrenia patients and the meta-analysis result remains the same if we withdraw our dataset. There are two potential factors for why drug-naive schizophrenia patients may have higher serum Pb. Personal habits such as hand washing before eating was suggested as a protective factor from elevated blood Pb level [30], while drug-naive schizophrenia patients are characterized by disorganized thinking and loss of goal-directed behaviors [7], which may lead to abnormal personal habits and higher levels of Pb in the blood. In addition, several studies indicated that patients with schizophrenia have a higher frequency of smoking [31], which may contribute to Pb exposure and the increased serum Pb level [32]. The mechanistic roles of Pb in schizophrenia and brain biological functions have been investigated in previous animal and epidemiological studies. First, Pb exerts an impact on the dopaminergic and glutamatergic systems and interferes with the normal function of neurotransmission [33], which are suggested as having a key role in the pathoetiology of schizophrenia [34]. Another known effect of Pb is induction of oxidative stress [35] and neurotoxicity by increasing the consumption of free radicals and activating apoptotic processes in astrocytes [27,35]. In addition, Pb has also been indicated to interact with etiological factors associated with schizophrenia, including the mutant form of DISC1 [12] and the DISC1-Binding Zinc-finger protein [17]. In our study, serum concentration of Pb was found to be negatively correlated with metabolic biomarkers dysregulated in schizophrenia (liver function (i.e. ALB) and renal function (i.e., BUN)). Both animal and clinical studies have suggested that lead-induced toxicity or oxidative stress could accumulate or affect liver and kidneys [36,37]. Hence, our study proposed that excess Pb may result in an increased risk of schizophrenia and play a role in liver and kidney metabolisms in schizophrenic patients at a drug-free condition.

It has been demonstrated that Cr is one of the major inorganic environmental pollutants, which affects human physiology and causes severe health problems such as inflammatory diseases, degenerative diseases and schizophrenia [38,39,40]. In the meta-analysis, the random-effect model derived from the five case-control studies suggested the concentration of Cr was associated with schizophrenia. However, under drug-free conditions, both our study and the meta-analysis consistently showed no association between Cr and schizophrenia. These results are consistent with two independent studies conducted in the Chinese population, where serum Cr concentration showed no association with schizophrenia under drug-free conditions [13] and showed association with schizophrenia under treatment condition [19]. Moreover, several studies have shown that Cr played a role in the treatment and alleviation of the side effects of antipsychotic drug in schizophrenia. For example, one study has suggested that Cr can be used as a treatment for psychiatry disease and have a positive impact on the patient’s mood by lowering the functional sensitivity of the serotonin 2A receptor [41]. In Yang’s study, daily intake of Cr supplements can improve the side-effect of anti-schizophrenia drugs such as weight gain and metabolic disorders [42]. Therefore, our results assume that serum Cr concentrations may correlate with antipsychotics; however, whether its concentration is associated with the risk of developing schizophrenia and/or the progress of schizophrenia will require further research.

Arsenic is one of the poisons which causes disorganization of the cytoskeletal framework, leading to As-induced neurotoxicity [11]. Both animal and clinical studies revealed that chronic As exposure could induce serious neurological effects and lead to neurobehavioral alterations [11,43]. Two previous studies conducted with non-drug-naive schizophrenic patients and healthy controls in the Chinese population suggested significant differences in As concentrations between the two groups but the trend is opposite (i.e., one study showed the concentration was higher in the cases [19] while the other reported that it was higher in the controls [18]). However, no significant association was observed between serum As concentration and the risk of schizophrenia in the study performed with drug naïve schizophrenic patients [13], which is consistent with both our study and the meta-analysis. On the other hand, studies have suggested that As is associated with liver and kidney problems [44,45]. In the present study, serum concentration of As is positively correlated with the metabolic biomarkers reflecting liver and renal functions (i.e., ALB, TP and BUN), which are also significantly different between schizophrenic patients and healthy controls. These results suggest that As may correlate with hepatotoxicity and nephrotoxicity in schizophrenic patients.

Limitations in the present study are addressed as follows. First, our data presented changes in THM concentrations in the peripheral blood and further research is needed to adequately determine whether such changes reflect relevant alterations in the human brain. Second, the possibility that genetic profiles play a role in schizophrenia in the patients [46] was not considered. However, the present study has several strengths in evaluating the associations between THMs and risk of schizophrenia. First, the quantification of four THMs followed the same experimental conditions and was performed on the same day, ensuring consistency in the experimental conditions. In addition, this is the first meta-analysis with case-control studies focusing on the association between THMs and schizophrenia.

## 5. Conclusions

Under drug-free conditions, high levels of Pb were associated with an elevated risk of schizophrenia. We also observed antipsychotic drug treatment as a confounding factor, where drug-naïve and drug treated patients show different results in the association between schizophrenia and THMs (Pb and Cr). Since antipsychotic drugs may interfere with serum THM concentrations, recruitment of drug-naïve patients or applying subgroup analysis on drug treatment is warranted for this type of study.

## Figures and Tables

**Table 1:** Distribution of the characteristics of schizophrenic patients (cases) and healthy subjects (controls).

Characteristics	Cases (*N* ^a^ = 95)	Controls (*N* = 95)	*p* ^b^
Age (years)			
<25	19 (20.0)	32 (33.7)	0.180
25–30	33 (34.7)	27 (28.4)	
30–35	22 (23.2)	16 (16.8)	
>35	21 (22.1)	20 (20.1)	
Sex			
Male	41 (43.2)	49 (51.6)	0.309
Female	54 (56.8)	46 (48.4)	
BMI (kg/m^2^)			
<18.5	5 (5.3)	2 (2.2)	0.719
18.5–24.9	57 (60.0)	57 (61.3)	
25–29.9	27 (28.4)	27 (29.0)	
≥30	6 (6.3)	7 (7.5)	
Marital status			
Single	73 (76.8)	45 (47.4)	<0.001
Married	22 (23.2)	50 (52.6)	
Education level			
Elementary school or below	23 (24.2)	25 (26.3)	0.305
Junior high school	43 (45.3)	42 (44.2)	
High school	16 (16.8)	22 (23.2)	
College or above	13 (13.7)	6 (6.3)	
Active smoking			
No	70 (73.7)	73 (76.8)	0.717
Yes	25 (26.3)	22 (23.2)	
Drinking			
No	88 (92.6)	82 (86.3)	0.237
Yes	7 (7.4)	13 (13.7)	

^a^ Number of subjects. ^b^ Pearson’s chi-square test.

**Table 2:** Serum concentrations of THMs in schizophrenic patients (cases) and healthy subjects (controls).

Metals (ng/mL)	Cases	Controls	*p* ^a^	Ratio (Cases/Controls)
Cr	0.850 (0.743–0.996)	0.852 (0.763–0.932)	0.770	0.998
Cd	0.756 (0.619–0.886)	0.768 (0.655–0.898)	0.405	0.984
Pb	0.626 (0.488–0.855)	0.546 (0.381–0.727)	0.011	1.15
As	0.593 (0.253–0.984)	0.767 (0.325–1.800)	0.025	0.773

^a^ In comparison with the median of controls by Mann-Whitney U test. THMs: toxic heavy metals.

**Table 3:** Associations between the prevalence of schizophrenia and the concentrations of four toxic heavy metals (THMs).

Toxic Metals	Median (IQR) ^a^	Univariate OR (95% CI) ^b^	*p*	Adjusted OR (95% CI)	*p* ^c^
Cr	0.851 (0.748–0.956)	0.65 (0.31–1.40)	0.276	0.74 (0.34–1.61)	0.452
Cd	0.762 (0.646–0.893)	0.64 (0.14–2.91)	0.562	0.59 (0.12–2.86)	0.508
Pb	0.607 (0.441–0.791)	3.21 (1.32–7.81)	0.010	3.15 (1.24–7.99)	0.016
As	0.604 (0.299–1.406)	0.85 (0.71–1.01)	0.070	0.87 (0.72–1.04)	0.118

^a^ IQR, inter-quartile range, unit: ng/mL. ^b^ Calculated by an unconditional Logistic regression model. ^c^ Adjusted OR and 95% CI were calculated by an unconditional logistic regression model adjusting for the potential confounders (i.e., marital status).

**Table 4:** Spearman correlations (rho) of PANSS score and concentration of THMs in schizophrenia patients.

EMEs	PANSS Score (Positive)	PANSS Score (Negative)	PANSS Score (General)	PANSS Score (Total)
Cr	−0.04	0.158	−0.117	−0.035
Cd	−0.008	0.030	0.011	0.068
Pb	0.150	0.288 **	0.006	0.324 **
As	0.103	−0.085	−0.017	0.012

* *p* < 0.05. ** *p* < 0.01. PANSS: The Positive and Negative Syndrome Scale.

**Table 5:** Distribution of the metabolic biomarker and their Spearman correlations (rho) with THMs

Metabolic Biomarker	Cases (*N* ^a^ = 95)	Controls (*N* = 95)	*p* ^b^	Correlations with THMs			
				Cr	Cd	Pb	As
Glucose metabolism							
FBG (mmol/L); median (IQR)	4.60 (4.30–5.20)	5.13 (4.71–5.73)	<0.001	−0.058	0.048	−0.023	0.039
Lipid metabolism							
TG (mmol/L); median (IQR)	1.44 (1.01–2.15)	1.32 (1.02–1.86)	0.455	0.134	0.136	0.065	0.066
TC (mmol/L); median (IQR)	4.13 (3.55–4.66)	4.90 (4.29–5.76)	<0.001	0.045	0.065	−0.100	0.186 *
Liver function							
AST (U/L); median (IQR)	17 (15–22)	17 (15–21)	0.610	0.059	−0.138	−0.076	0.002
ALT (U/L); median (IQR)	18 (13–29)	15 (12–20)	0.006	−0.054	−0.132	−0.080	0.046
ALB (g/L); median (IQR)	40.00 (38.00–43.00)	42.12 (39.46–44.89)	<0.001	0.012	0.054	−0.163 *	0.163 *
TP (g/L); median (IQR)	68.00 (64.00–71.00)	69.66 (64.07–76.39)	0.021	0.106	0.100	−0.038	0.208 **
Renal function							
BUN (mmol/L); median (IQR)	4.08 (3.37–5.19)	5.24 (4.46–6.45)	<0.001	0.028	−0.042	−0.182 *	0.217 **
CREA (umol/L); median (IQR)	65.00 (57.00–72.00)	64.05 (54.13–73.18)	0.725	0.075	−0.052	−0.207 **	0.014
UA (umol/L); median (IQR)	283.00 (217.00–353.00)	262.00 (221.75–369.25)	0.943	−0.014	−0.094	−0.170 *	0.076

^a^ Number of subjects. ^b^ In comparison with the median of controls by Mann-Whitney U test. * *p* < 0.05. ** *p* < 0.01. THMs: toxic heavy metals.

## Supplementary Materials

The following are available online at https://www.mdpi.com/1660-4601/16/21/4200/s1, Table S1. Measured and standard concentrations of the certified reference materials; Table S2. Limit of detection and reproducibility of four THMs; Table S3. Main characteristics of included studies in the meta-analysis; Table S4. Results of meta-analysis of 4 THMs; Figure S1: Forest plot of subgroup analysis stratified by whether the patients with schizophrenia treated with antipsychotic drugs (without present study). Overall pooled prevalence was calculated by random-effects module. Horizontal bars show 95% CIs. drug = 1, patients with schizophrenia were treated with antipsychotic drugs. drug = 0, patients with schizophrenia were not treated with antipsychotic drugs; Figure S2. Sensitivity analysis through deletion of one study at a time to reflect the influence of the individual dataset to the pooled SMDs of the THM concentrations in patients with schizophrenia and healthy controls.
