# Supplementary material for: Association of Typical Toxic Heavy Metals with Schizophrenia
Source: Int J Environ Res Public Health. 2019 Oct 30;16(21):4200. doi: 10.3390/ijerph16214200 (PMC6862006; doi:10.3390/ijerph16214200)
Supplement: Supplementary file 1 [file ijerph-16-04200-s001.pdf]

Supplementary Materials

Table S1. Measured and standard concentrations of the certified reference materials.

THMs	Measured Value [a] (ng/mL)	Standard Value [b] (ng/mL)	Reference Type	Coincidence (%)
Cr	6.76 ± 0.13	6.99 (5.24–8.74)	Serum [c]	96.76
Cd	4.95 ± 0.09	4.92 (3.94–5.90)	Serum [c]	100.59
Pb	3.68 ± 0.03	3.83 ± 0.18	Hair [d]	101.03
As	19.90 ± 0.55	19.70 (15.80–23.60)	Serum [c]	95.98

[a] The measured value is the mean and standard deviation for three repeated measurements. [b] Standard values are provided by the company. c ClinChek® Serum Controls (trace elements level II, REF 8881, EVISA). [d] Human Hair Certified Reference Material (GBW09101b, China).

Table S2. Limit and precision of detection for four toxic heavy metals (THMs).

THMs	Instrument Detection Limit (ng/mL)	Dilution Factor	Method Detection Limit (ng/mL)	Repeatability Rel. Repeat. SD [#]	Reproducibility Rel. Repro. SD [*]
**Cr**	**0.002**	**20**	0.04	1.86%	2.38%
Cd	0.003	20	0.06	1.88%	2.32%
Pb	0.010	20	0.20	0.89%	2.24%
As	0.016	20	0.32	2.77%	3.70%

[#] Relative repeatability standard deviation: calculated by extracting 3 measurements from same day. [*] Relative reproducibility standard deviation: calculated by extracting 3 measurements from 3 different days.

Table S3. Main characteristics of included studies in the meta-analysis.

No.	Author[a]	Drug[b]	Published year	Area	Geographic area	Sample type	Sample size Case	Sample size control	THMs	NOS score
[1]	Guo et al.	1	2013	China	Asia	serum	119	126	Cr, Cd, Pb, As	9
[2]	Arinola et al._1	0	2010	Nigeria	Africa	plasma	15	20	Cr, Cd, Pb	7
	Arinola et al._2	1	2010	Nigeria	Africa	plasma	20	20	Cr, Cd, Pb	7
[3]	Cai et al._1	0	2015	China	Asia	serum	61	61	Cr, Cd, Pb, As	8
	Cai et al._2	0	2015	China	Asia	serum	50	49	Cr, Cd, Pb, As	8
[4]	Li et al.	1	2018	China	Asia	serum	158	669	Cr, As	8

[5]	P.C. Stanley et al._1	0	2002	Nigeria	Africa	serum	20	20	Cd, Pb	6
	P.C. Stanley et al._2	1	2002	Nigeria	Africa	serum	20	20	Cd, Pb	6
	P.C. Stanley et al._3	1	2002	Nigeria	Africa	serum	20	20	Cd, Pb	6
[6]	Present study	0	-	China	Asia	serum	95	95	Cr, Cd, Pb, As	9

[a] Arinola et al._1 The schizophrenic patients were newly diagnosed and not taking antipsychotic drugs. Arinola et al._2 The schizophrenic patients had used antipsychotic drugs for at least 2 weeks. Cai et al._1 The case and control were from a training set. Cai et al._2 The case and control were from a test set. P.C. Stanley et al._1 The schizophrenic patients before the start of treatment. P.C. Stanley et al._2 The schizophrenic patients at 3 weeks after treatment. P.C. Stanley et al._3 The schizophrenic patients at 7 weeks after treatment. [b] 0: patients with schizophrenia were not treated with antipsychotic drugs**.** 1: patients with schizophrenia were treated with antipsychotic drugs.

Table S4. Results of meta-analysis of 4 THMs.

THMs	Groups	No. of studies	Sample size		Test of association		Test of heterogeneity	
			Case	Control	SMD	95%CI	I^2	p
Cr	Overall	5	518	1020	0.3246	0.0166–0.6326	83.0%	<0.01
	Drug = 0	3	211	225	0.3134	−0.2770–0.9038	88.2%	0.7640
	Drug = 1	3	297	815	0.4063	0.2662–0.5464	0.0%	
Cd	Overall	6	538	1040	−0.0357	−0.4812–0.4098	92.6%	<0.01
	Drug = 0	4	241	245	0.2796	−0.2488–0.8079	86.2%	0.0978
	Drug = 1	4	317	835	−0.3763	−0.9452–0.1927	91.1%	
Pb	Overall	6	538	1040	0.2537	−0.2108–0.7183	93.5%	<0.01
	Drug = 0	4	241	245	0.4064	0.1371–0.6757	47.9%	0.3337
	Drug = 1	4	317	835	0.0240	−0.7033–0.7512	95.0%	
As	Overall	4	483	1000	0.4459	−1.1994–2.0912	99.4%	<0.01
	Drug = 0	2	206	205	−0.1397	−0.3889–0.1095	99.8%	0.4560
	Drug = 1	2	277	795	1.2972	−2.4727–5.0672	37.4%	

Reference

1. Guo, J.; Zeng, J.; Yan, L.L.; Qiu, J.Y.; Feng, F.B.; Wang, J.Y. Quantitative detection and analysis of 41 kinds of trace elements in serum of patients with schizophrenia. *Clin J Med Offic*. **2013**, *41*, 44–26.
2. Arinola, G., B.; Idonije, K.; Akinlade, and O. Ihenyen. Essential trace metals and heavy metals in newly diagnosed schizophrenic patients and those on anti-psychotic medication. *J Res Med Sci.* **2010**, *15*, 245–9.
3. Cai, L., T.; Chen, J.; Yang, K.; Zhou, X.; Yan, W.; Chen, L.; Sun, L.; Li, S.; Qin, P.; Wang, et al. Serum trace element differences between Schizophrenia patients and controls in the Han Chinese population. *Sci Rep*. **2015,** *5*, 15013.
4. Li, Z.; Liu, Y.M.; Li, X.; Ju, W. Wu, G.R.; Yang, X.M.; Fu, X.F. Gao, X.B. Association of Elements with Schizophrenia and Intervention of Selenium Supplements. *Biol Trace Elem Res*. **2018,** *183*, 16–21.

5. Stanley, P.C.; V.C. Wakwe. Toxic trace metals in the mentally ill patients. *Niger Postgrad Med J.* **2002**. *9*, 199–204.

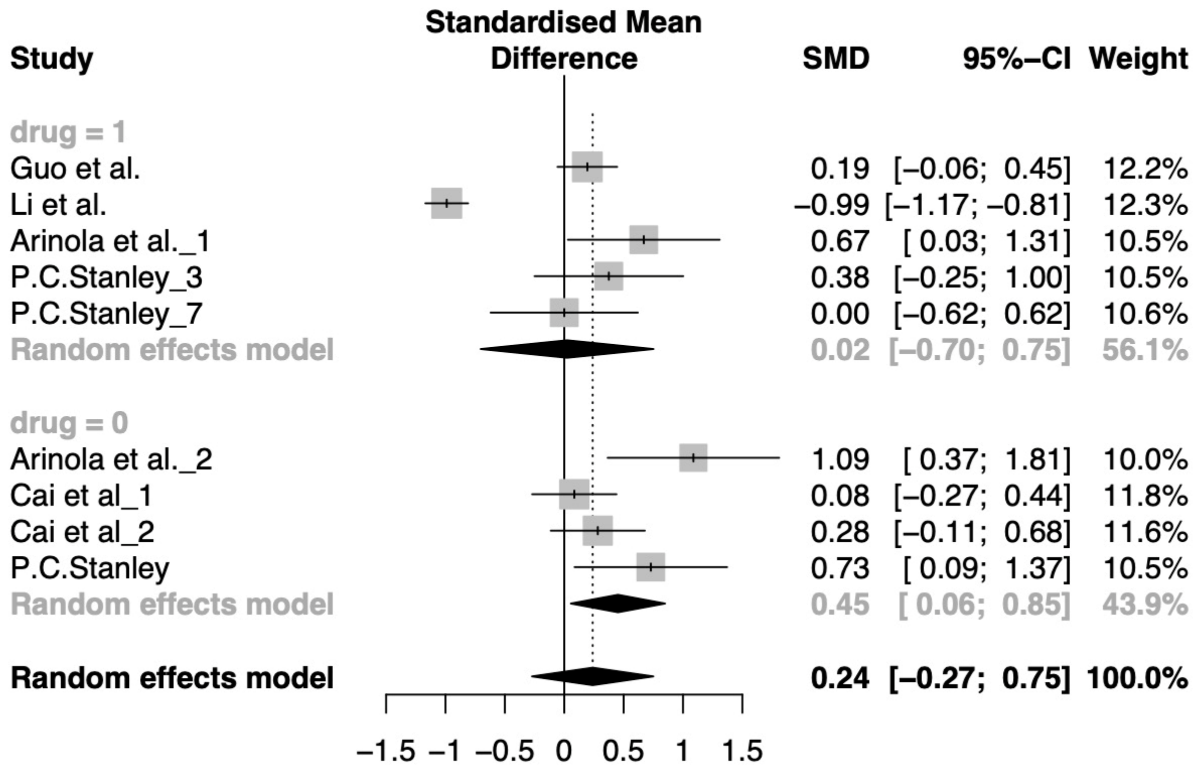

Figure S1. Forest plot of subgroup analysis stratified by whether the patients with schizophrenia treated with antipsychotic drugs (without present study). Overall pooled prevalence was calculated by random-effects module. Horizontal bars show 95% CIs. drug = 1, patients with schizophrenia were treated with antipsychotic drugs. drug = 0, patients with schizophrenia were not treated with antipsychotic drugs.

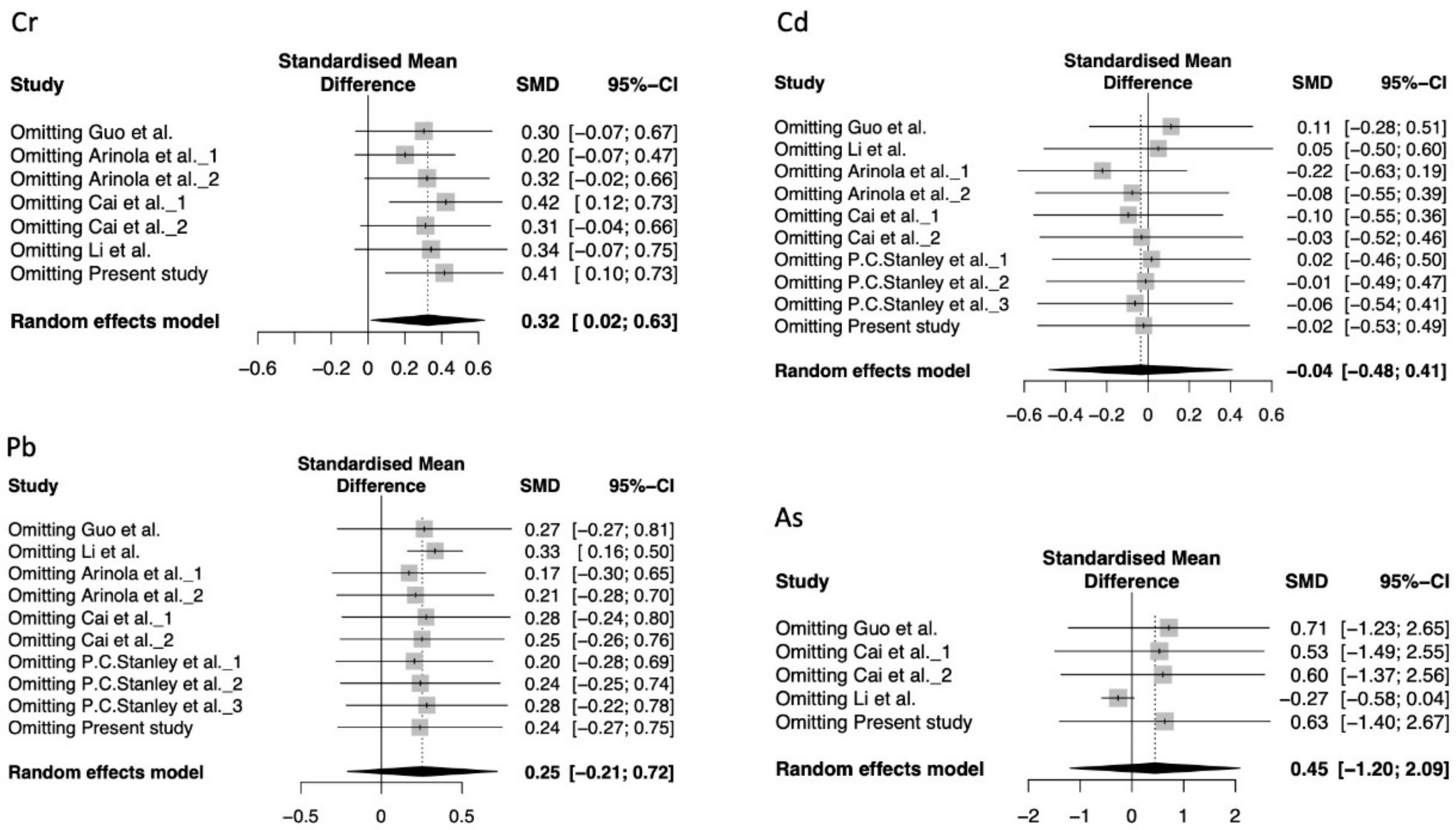

Figure S2. Sensitivity analysis through deletion of one study at a time to reflect the influence of the individual dataset to the pooled SMDs of the THM concentrations in patients with schizophrenia and healthy controls.
